# C-terminal α-synuclein truncations are linked to cysteine cathepsin activity in Parkinson's disease

**DOI:** 10.1074/jbc.RA119.008930

**Published:** 2019-05-15

**Authors:** Ryan P. McGlinchey, Shannon M. Lacy, Katherine E. Huffer, Nahid Tayebi, Ellen Sidransky, Jennifer C. Lee

**Affiliations:** From the ‡ Laboratory of Protein Conformation and Dynamics, Biochemistry and Biophysics Center, National Heart, Lung, and Blood Institute and; § Medical Genetics Branch, National Human Genome Research Institute, National Institutes of Health, Bethesda, Maryland 20892

**Keywords:** amyloid, alpha-synuclein (α-synuclein), lysosome, mass spectrometry (MS), electron microscopy (EM), transmission electron microscopy, Lewy body, cysteine cathepsin, proteolytic processing, protein aggregation

## Abstract

A pathological feature of Parkinson's disease (PD) is Lewy bodies (LBs) composed of α-synuclein (α-syn) amyloid fibrils. α-Syn is a 140 amino acids–long protein, but truncated α-syn is enriched in LBs. The proteolytic processes that generate these truncations are not well-understood. On the basis of our previous work, we propose that these truncations could originate from lysosomal activity attributable to cysteine cathepsins (Cts). Here, using a transgenic *SNCA*^A53T^ mouse model, overexpressing the PD-associated α-syn variant A53T, we compared levels of α-syn species in purified brain lysosomes from nonsymptomatic mice with those in age-matched symptomatic mice. In the symptomatic mice, antibody epitope mapping revealed enrichment of C-terminal truncations, resulting from CtsB, CtsL, and asparagine endopeptidase. We did not observe changes in individual cathepsin activities, suggesting that the increased levels of C-terminal α-syn truncations are because of the burden of aggregated α-syn. Using LC-MS and purified α-syn, we identified C-terminal truncations corresponding to amino acids 1–122 and 1–90 from the *SNCA*^A53T^ lysosomes. Feeding rat dopaminergic N27 cells with exogenous α-syn fibrils confirmed that these fragments originate from incomplete fibril degradation in lysosomes. We mimicked these events *in situ* by asparagine endopeptidase degradation of α-syn fibrils. Importantly, the resulting C-terminally truncated fibrils acted as superior seeds in stimulating α-syn aggregation compared with that of the full-length fibrils. These results unequivocally show that C-terminal α-syn truncations in LBs are linked to Cts activities, promote amyloid formation, and contribute to PD pathogenesis.

## Introduction

**Figure 1.**
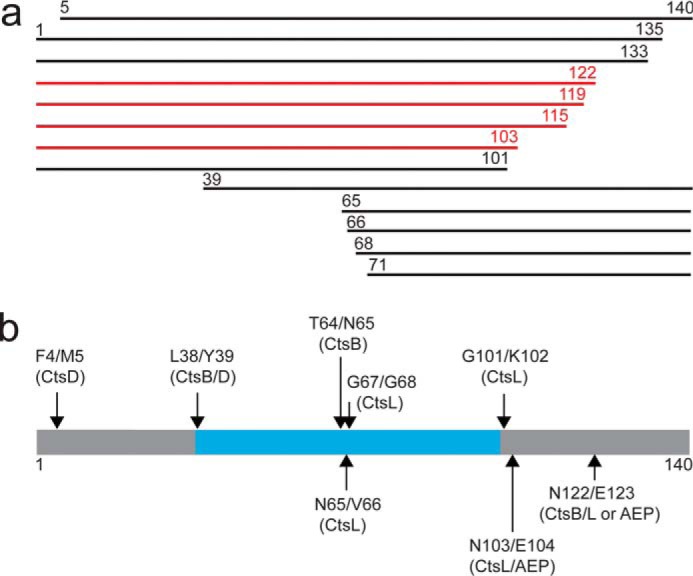
**Linking α-synuclein truncations found in PD to lysosomal activity.** *a*, α-Syn truncations identified by MS from brain tissues (12, 13) and enriched LBs (2) from PD patients. C-terminal α-syn truncations observed from at least two independent studies are colored *red. b*, schematic representation of α-syn with cleavage sites (denoted by *arrows*) derived from lysosomal degradation of α-syn (27) that would result in fragments observed in LBs. Cleavage sites are assigned to individual lysosomal cathepsins D (CtsD), B (CtsB), L (CtsL), and asparagine endopeptidase (AEP) (27). The polypeptide region (about residues 37–99) found in the β-sheet amyloid fibril core determined by cryo-EM studies is colored *cyan* (68–70).

A hallmark of Parkinson's disease (PD)^2^ is the presence of Lewy bodies (LBs), intracytoplasmic inclusion bodies rich in α-synuclein (α-syn) amyloid fibrils (1). Various posttranslational modifications of α-syn, including phosphorylation (2), ubiquitination (3), oxidation (4), and truncations, are associated with PD (5). Ordinarily α-syn is an 140 amino acid protein, but a significant amount of α-syn in Lewy bodies is truncated (6), lacking either the C- or N-terminal region. Understanding the proteolytic processes that result in these truncations is an active area of research (7–11, 71). Several α-syn truncations have been identified in samples from PD patients by MS (Fig. 1*a*), including those comprising segments 5–140, 1–135, 1–133, 1–122, 1–119, 1–115, 1–103, 1–101, 39–140, 65–140, 66–140, 68–140, and 71–140 (2, 12, 13). Although little work has been done on the impact of N-terminal residues on the propensity of α-syn to aggregate (14, 15), it is well-established that C-terminal truncated variants accelerate amyloid formation *in vitro* (16–20) and in cellular models (21, 22). Thus, the presence of these aggregation-prone truncations may promote fibril formation, contributing to disease progression. Indeed, attempts to reduce C-terminal truncations by immunotherapy in PD mouse models have shown promising results (23).

Understanding the degradation processes that generate C-terminal truncations (ΔC) would aid in the elucidation of new ways to circumvent the progression of PD. Mounting evidence supports the involvement of the lysosome and proteasome in α-syn degradation (24, 25). However, because the lysosome is generally considered to be responsible for removal of aggregation-prone species, we hypothesize that these truncations stem from incomplete proteolytic events in this organelle. In fact, the lysosomal protease, cathepsin D (CtsD) was shown to generate α-synΔC species (26, 27). More recently, the lysosomal cysteine cathepsin asparagine endopeptidase (AEP), found to be elevated in PD brains, was reported to generate an α-syn fragment composed of residues 1–103, which enhanced neurotoxicity in a PD mouse model (28). Although our interests are in a lysosomal role in generating ΔC-terminal α-syn truncations, cytosolic proteases such as calpain-I (7–9), caspase-1 (10) and neurosin (11) have also been considered in generating C-terminal truncations.

Based on our prior work detailing a complete peptide map of the lysosomal degradation of α-syn (27), we suggest that many of these truncated forms in LBs could arise because of incomplete degradation by cysteine and aspartyl cathepsins. Specifically, cleavage sites at Phe-4/Met-5, Leu-38/Tyr-39, Thr-64/Asn-65, Asn-65/Val-66, Gly-67/Gly-68, Gly-101/Lys-102, Asn-103/Glu-104, and Asn-122/Glu-123 (where a *shill* indicates the cut site) (Fig. 1*b*) could generate the corresponding fragments 5–140, 39–140, 65–140, 66–140, 68–140, 1–101, 1–103, and 1–122, respectively. Both aspartyl CtsD and cysteine cathepsins B (CtsB), L (CtsL), and AEP can generate these peptides, with the majority attributable to cysteine cathepsins (27, 29). Other cleavage sites are assigned to specific cathepsins such as Phe-4/Met-5 (CtsD), Thr-64/Asn-65 (CtsB), Asn-65/Val-66 (CtsL), Gly-67/Gly-68 (CtsL), and Gly-101/Lys-102 (CtsL). Although it remains to be determined whether these truncations arise from incomplete lysosomal degradation of α-syn under disease-related conditions, there appears to be a connection between cysteine cathepsin activity and PD. Patients with PD have increased levels of CtsL within nigral neurons (30). The ubiquitously expressed CtsB was found to induce α-syn aggregate formation originating in the lysosome (31). In addition, the *CTSB* gene was identified as a PD risk allele (32), and elevated CtsB activity was recently reported in dementia with LBs (DLB), another synucleinopathy (33).

In this work, we sought to determine which α-syn truncations found in LBs are lysosomal in origin. Lysosomes were purified from two disease-related models: brains from transgenic mice overexpressing the PD-associated A53T mutant form of α-syn (*SNCA*^A53T^) (34) and cultured N27 rat dopaminergic neuronal cells treated with fibrils formed *in vitro* by N-terminally acetylated α-syn (hereafter, simply abbreviated as α-syn). Aged *SNCA*^A53T^ develops hind leg paresis, weight loss, and difficulty ambulating, leading to progressive neurodegeneration and death, but the actual age of disease onset varies. Here, we compared symptomatic *SNCA*^A53T^ mice to age-matched nonsymptomatic *SNCA*^A53T^ mice as well as nontransgenic (*wt*) control mice. Using antibody epitope mapping, we show that C-terminal truncations (corresponding to 12- and 8-kDa bands on Western blots) were enhanced only when the mice became symptomatic. Interestingly, in the second N27 model, two bands of similar weight were also observed after partial digestion of preformed fibrils in lysosomes. The identities of the α-synΔC species were determined using recombinant α-syn and peptide mapping by MS. Selective inhibition studies verified the involvement of CtsB, CtsL, and AEP in generating the 12-kDa band, whereas only CtsB contributed to produce the 8-kDa band. To better understand the mechanism involved, we recapitulated these proteolysis events *in situ* by AEP degradation of preformed α-syn fibrils. Importantly, the AEP-derived 1–122 and 1–103 fibrils stimulated aggregation of soluble full-length α-syn. These data unequivocally show that α-synΔC species in LBs are linked to cysteine cathepsin activities and serve as potent amyloid seeds. Collectively, this work demonstrates a new molecular connection between the lysosome and PD pathology.

## Results

### α-SynΔC species are enriched in lysosomes isolated from symptomatic SNCA^A53T^ mice

**Figure 2.**
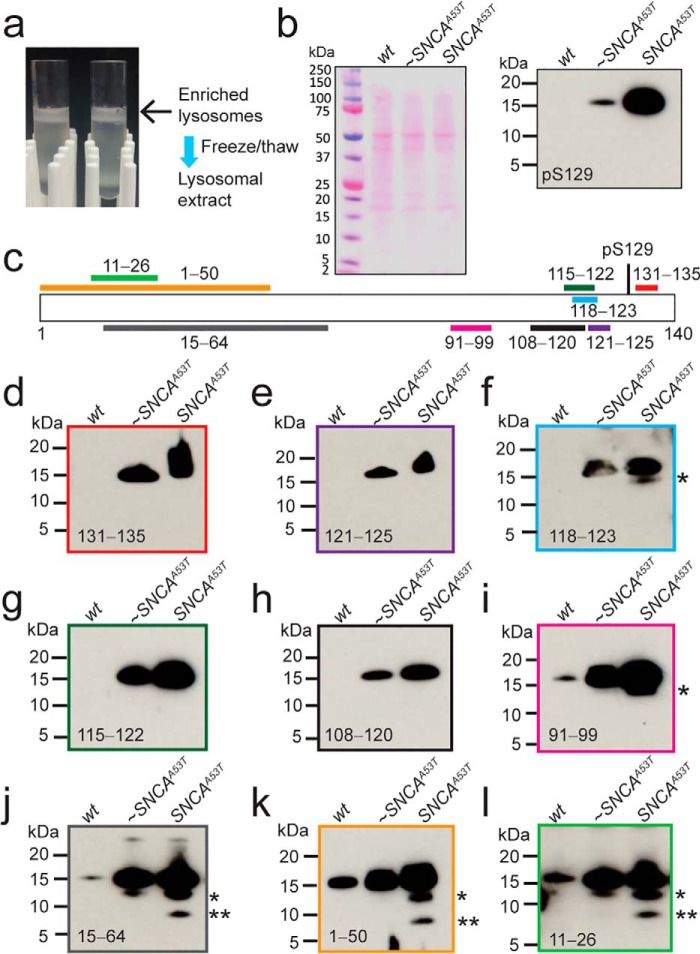
**Comparison of α-syn levels in lysosomes isolated from mouse brains.** *a*, image of enriched lysosomal fractions after density gradient centrifugation. To generate lysosomal extracts, samples were frozen/thawed several times. *b*, *left*, Ponceau–stained PVDF membrane of lysosomal extracts isolated from age-matched nontransgenic (*wt*), nonsymptomatic (∼*SNCA*^A53T^) and symptomatic (*SNCA*^A53T^) mice. *b*, *right*, Western blot analysis of lysosomal extracts probed with Ser-129–phosphorylated α-syn antibody. *c*, schematic representation of α-syn sequence indicating epitope regions of antibodies used to detect full-length and truncated forms of α-syn from lysosomal extracts. *d–k* and *m*, lysosomal extracts probed with antibodies against α-syn at residues 131–135 (*d*), 121–125 (*e*), 118–123 (*f*), 115–122 (*g*), 108–120 (*h*), 91–99 (*i*), 15–64 (*j*), 1–50 (*k*), and 11–26 (*l*).

Mice overexpressing human *SNCA*^A53T^ were euthanized when symptoms developed, typically at ∼16 months of age (34). Samples from age-matched nonsymptomatic (∼*SNCA*^A53T^) as well as nontransgenic (*wt*) mice were compared with those from the symptomatic mice. Lysosomes were isolated and enriched from mouse brain samples by density gradient centrifugation and lysosomal extracts were prepared by repeated freeze-thaw cycles (Fig. 2*a*). Immediately after generating lysosomal extracts, we probed for the presence of endogenous α-syn in lysosomes to capture levels of undigested α-syn. First, the presence of Ser-129–phosphorylated α-syn, a hallmark of LBs (35), was evaluated, and lysosomes from symptomatic *SNCA*^A53T^ mice were found to have dramatically more Ser-129–phosphorylated α-syn than nonsymptomatic ∼*SNCA*^A53T^ littermates (Fig. 2*b*), indicating that α-syn is hyperphosphorylated only in a disease-state (35). Interestingly, lysosomes from *wt* mice showed no immunoreactivity toward the Ser-129 antibody, even though the epitope region is conserved between murine and human sequences (Fig. S1).

The state of α-syn in lysosomes was evaluated using an array of antibodies that have defined epitopes in the N and C terminus (Fig. 2*c*). Lysosomal extracts from *wt*, ∼ *SNCA*^A53T^, and *SNCA*^A53T^ mice were probed with C-terminal epitopes against human α-syn at residues 131–135 (Fig. 2*d*), 121–125 (Fig. 2*e*), 118–123 (Fig. 2*f*), 115–122 (Fig. 2*g*), and 108–120 (Fig. 2*h*). Western blots showed enhanced levels of full-length α-syn in symptomatic *versus* nonsymptomatic *SNCA*^A53T^ mice, with none detected in *wt* samples. The lack of reactivity for endogenous α-syn can be attributed to amino acid differences between murine and human α-syn in the C terminus (Fig. S1). Specifically, residues Ala-107, Asp-121, and Asn-122 in human correspond to Tyr-107, Gly-121, and Ser-122 in murine α-syn, making these C-terminal antibodies highly specific for the human sequence.

The antibody that recognizes epitope 118–123 of α-syn (Fig. 2*f*) showed an additional band at 12-kDa (denoted by an *asterisk*) in lysosomal samples from symptomatic *SNCA*^A53T^ mice. Because this band was not present using all other C-terminal antibodies (Fig. 2, *d*, *e*, *g*, and *h*), it can be inferred that identity of this C-terminal truncation results from a cleavage site around residues 118–123. Analysis with an antibody that recognizes an epitope at residues 91–99 of human and murine α-syn (Fig. 2*i*) also consistently showed this 12-kDa band in lysosomes from the symptomatic mice.

Antibodies specific for the N-terminal region (residues 1–64) of α-syn revealed an 8-kDa band (Fig. 2, denoted by two *asterisks*) in addition to the 12-kDa band. Specifically probing with antibodies that recognize epitope regions 15–64 (Fig. 2*j*), 1–50 (Fig. 2*k*), and 11–26 (Fig. 2*l*) demonstrated that truncations were more abundant in lysosomes from *SNCA*^A53T^ mice. Because this band was not observed by the antibody that recognizes epitope 91–99, the protein is likely cleaved N-terminal to residue 99 and likely contains the majority of the N terminus. The N-terminal sequence of α-syn is highly conserved between humans and mice except for two amino acid differences at positions 53 and 87 (Fig. S1). The lack of truncated forms in *wt* lysosomes suggests that the degradation pattern of murine α-syn differs from the human form. Clearly, these data show not only an increase in full-length α-syn but an enrichment in α-synΔC species in lysosomes from symptomatic *SNCA*^A53T^ mice.

### Evaluation of cathepsin activities in lysosomes isolated from SNCA^A53T^ mice

**Figure 3.**
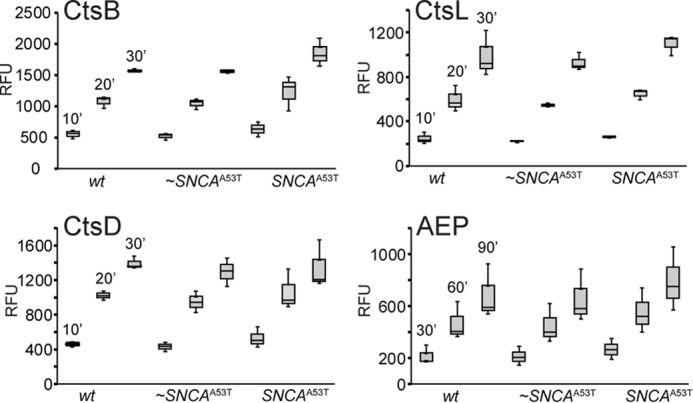
**Comparison of cathepsin activities in lysosomes isolated from *SNCA*^A53T^ mice.** Endogenous protease activities in lysosomal extracts from *wt*, nonsymptomatic (∼*SNCA*^A53T^), and symptomatic (*SNCA*^A53T^) mice measured by fluorogenic substrates. Ac-RR-AMC for CtsB, Ac-FR-AMC for CtsL, MCA-GKPILEFRKL(Dnp)-D-R-NH_2_ for CtsD, and AENK-AMC for AEP were incubated with lysosomal extracts (1 μg total protein) at pH 5 with 5 mm DTT and 37 °C. Fluorescence was recorded as a function of time (10 to 90 min) and relative fluorescence units (*RFU*) are reported (*n* = 3). Data for the second set of biological replicates is shown in Fig. S2.

To assess specific lysosomal protease activity, fluorogenic substrates specific for CtsB, CtsL, CtsD, and AEP activity were used. Two biological replicates of lysosomal extracts (1 μg) from *wt*, ∼ *SNCA*^A53T^, and *SNCA*^A53T^ mice were incubated in the presence of fluorogenic substrates (Ac-RR-AMC for CtsB, Ac-FR-AMC for CtsL, MCA-GKPILEFRKL(Dnp)-D-R-NH_2_ for CtsD, and AENK-AMC for AEP) at pH 5 (Fig. 3). Analysis of individual activities as a function of time showed no significant changes in activity when mice became symptomatic. One exception, seen only in one dataset (Fig. S2) yet not in the other (Fig. 3), was an elevation of CtsB activity in lysosomes from ∼*SNCA*^A53T^ and *SNCA*^A53T^ mice. Additional biological replicates are needed to determine the significance of this observation. Nevertheless, the data indicate that it is not the loss of protease activity in lysosomes from symptomatic mice but rather the overburden of α-syn levels that is responsible for the incomplete degradation and the appearance of α-synΔC.

### Identification of α-synΔC species

**Figure 4.**
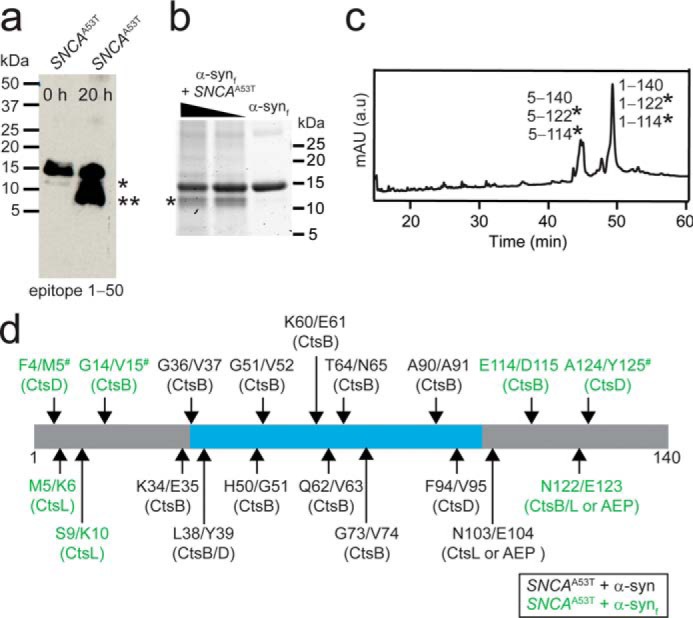
**Identification of cysteine cathepsins in generating α-syn truncations.** *a*, Western blot analysis of *SNCA*^A53T^ lysosomes probed with an N-terminal antibody (epitope 1–50) before (0 h) and after (20 h) incubation at room temperature. Bands migrating at 12 (*) and 8 kDa (**) were detected. *b*, degradation of preformed α-syn fibrils (α-syn_f_, 15 μm) by lysosomal extracts (10 and 30 μg total protein) from *SNCA*^A53T^ mice after 20 h at pH 5 and 37 °C assessed by SDS-PAGE (4–12%) and visualized by Coomassie Blue staining. *Asterisk* denotes a 12-kDa band. *c*, corresponding LC trace monitored at 210 nm of α-syn_f_ (15 μm) incubated with lysosomes (30 μg total protein) from *SNCA*^A53T^ mice after 20 h at pH 5 and 37 °C. *mAU*, milli absorbance units. Peptide fragments found in the corresponding peaks by MS analysis are listed in the order of their peptide abundance. *Asterisk* denotes assignment of 12 kDa band observed in *panels a* and *b*. All other assigned masses are reported in Table S1. *d*, schematic representation of the primary amino acid sequence of α-syn with identifiable cleavage sites generated from degradation of soluble α-syn (*black*) and α-syn_f_ (*green*) by lysosomes from *SNCA*^A53T^mice. Cutting that occurs in both soluble and fibrillar α-syn is indicated by #. Cathepsin(s) responsible for each cleavage site is as indicated based on this and prior work (27, 29). MS analyses are reported in Tables S1 and S2. Amyloid core (residues ∼37–99) determined by cryo-EM (68–70) is highlighted in *cyan*.

By monitoring degradation of the remaining endogenous α-syn in lysosomes from *SNCA*^A53T^ mice as a function of time, it was clear that the 12- and 8-kDa bands originated from full-length α-syn (Fig. 4*a*). Enrichment of these truncations after a 20-h incubation at pH 5 affirmed that these lysosomal extracts were active and continued to degrade endogenous α-syn over time. Because levels of endogenous α-syn could not be detected by MS under these experimental conditions, we turned to the use of preformed α-syn fibrils (Fig. S3) to identify these truncations. Fibrillar material was chosen because 1) Ser-129–phosphorylated α-syn, a well-accepted posttranslational modification for aggregated α-syn (35), is highly enriched in *SNCA*^A53T^ lysosomes (Fig. 2*b*) and 2) prior work on α-syn_f_ degradation by CtsL established that both the N- (residues 1 to 9) and C-terminal region (residues 101 to 140) are the most protease-sensitive regions of the fibril structure (29).

The resulting SDS-PAGE analysis revealed the formation of a 12-kDa band after incubation with α-syn fibrils at pH 5 for 20 h at 37 °C (Fig. 4*b*), reminiscent of that observed by Western blot analysis with endogenous α-syn (Fig. 4*a*). LC-MS analysis identified the 12-kDa species to be C-terminal truncations with cleavage sites at Glu-114/Asp-115, Asn-122/Glu-123, Ala-124/Tyr-125, and Glu-139/Ala-140 (Fig. 4*c*; Table S1). Based on the peptide abundance, the 1–122 fragment constitutes the main species. Furthermore, N-terminal truncations at Phe-4/Met-5, Met-5/Lys-6, Ser-9/Lys-10, and Gly-14/Val-15 were also present in smaller quantities. Under these conditions, a mass corresponding to the 8-kDa band could not be readily detected.

To figure out the identity of the 8-kDa band, the degradation pattern of soluble α-syn was evaluated. Upon incubation of α-syn with lysosomal extracts, one main species with molecular mass ∼8 kDa was detected by SDS-PAGE and Coomassie Blue staining (Fig. S4*a*). Corresponding LC-MS analysis suggested this mass is likely peptide fragment 1–90 (Fig. S4*b*). Aside from the cleavage site at Ala-90/Ala-91, other minor peptides with cleavage sites at Phe-4/Met-5, Gly-14/Val-15, Lys-34/Glu-35, Gly-36/Val-37, Leu-38/Tyr-39, His-50/Gly-51, Gly-51/Val-52, Lys-60/Glu-61, Gln-62/Val-63, Thr-64/Asn-65, Phe-94/Val-95, Asn-103/Glu-104, and Ala-124/Tyr-125 were identified (Fig. 4*d*; Table S2). Notably, 5–140, 65–140, and 1–103 truncations found in LBs were observed (Fig. 1*a*).

### Cysteine cathepsins are responsible for α-synΔC generation

Next, we asked which protease(s) are responsible for generating α-synΔC species by using the following selective lysosomal protease inhibitors: pepstatin A (PePA) for CtsD, CA-074 for CtsB, z-FY-DMK for CtsL, and z-AAN-AMC for AEP. Limited proteolysis experiments of α-syn_f_ with *SNCA*^A53T^ lysosomal extracts preincubated with individual inhibitors showed that none of the inhibitors significantly affected formation of the 12-kDa band (Fig. S5). LC-MS analysis (Table S3) showed that when selectively inhibiting either CtsB or CtsL, a loss in activity for one could be rescued by the other, because of a shared substrate site, cutting α-syn_f_ at Asn-122/Glu-123 to generate the peptide fragment 1–122. The other two C-terminal truncations at residues 114 and 124 were attributed to CtsB and CtsD, respectively (Fig. 4*d*). N-terminal cleavage sites were also assigned (27): Phe-4/Met-5 to CtsD, Met-5/Lys-6 and Ser-9/Lys-10 to CtsL, and Gly-14/Val-15 to CtsB activities, respectively.

For the 8-kDa band, selective inhibition experiments confirmed a complete absence of fragment 1–90 with the CtsB inhibitor CA-074, whereas PePA (CtsD inhibitor) significantly reduced this truncation (Fig. S4*a*). Although only Phe-4/Met-5 and Phe-94/Val-95 were inhibited with PePA, all other activities were inhibited by CA-074 as characterized by LC-MS (Table S4). Cleavage at site Ala-90/Ala-91 is unique to CtsB activity (27); thus, its reduction in the presence of a CtsD inhibitor requires a different interpretation to reconcile this observation. One scenario implies a more indirect role for CtsD activity, where CtsD directly activates CtsB (36, 37), which is then responsible for the generation of fragment 1–90. Alternatively, CtsD can first cleave α-syn at residue Phe-94 to generate the fragment 1–94, which could be a preferred substrate over full-length α-syn for CtsB activity.

### α-SynΔC species identified in lysosomes isolated from N27 rat dopaminergic cells

Motivated by our limited lysosomal degradation results showing that the 12-kDa α-synΔC species is derived from fibrillar α-syn, we sought to recapitulate this observation in cultured neuronal cells. Here, we asked whether α-syn truncations are generated after fibrils are endocytosed and trafficked to lysosomes in live cells (38–40). We chose immortalized N27 rat cells because they are dopaminergic (41). Cells were treated with both soluble and fibrillar α-syn at low and high protein concentrations (100 nm and 1 μm). To determine an appropriate treatment time frame, we used live cell imaging to evaluate cell viability. Over the course of 60-h treatment, only the cells treated with fibrils showed any noticeable effects. Fewer cells were observed, with many losing their dendritic morphology, and eventually disintegrating over time (Fig. S6*a*).

To verify uptake, Western blot analysis was performed on cells collected 48 h post treatment. Using a N-terminal epitope antibody, no bands were detected from cells treated with soluble α-syn, but both the full-length and a 12-kDa band were observed after treating with fibrils at the low and high concentrations (Fig. S6*b*). A series of higher molecular mass species were also present, indicating an aggregated state of α-syn. After treatment with soluble α-syn, little immunoreactivity was observed, likely because of its rapid degradation in N27 cells.

**Figure 5.**
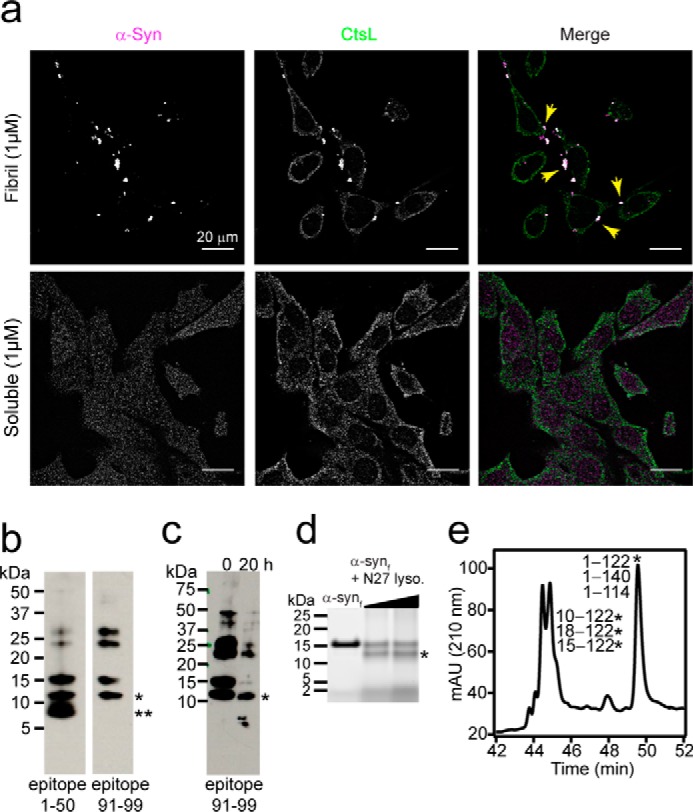
**C-terminal α-syn truncations directly derived from lysosomal activity.** *a*, immunofluorescence images of cultured N27 rat dopaminergic cells after a 48-h incubation with fibrillar (*top*) or soluble (*bottom*) α-syn (1 μm). α-Syn (*magenta*) was stained with an N-terminal (epitope 1–50) rabbit mAb, whereas endogenous CtsL (a lysosomal marker, *green*) was detected using a mouse mAb. *Arrows* highlight examples of colocalization. *b*, Western blot analysis of enriched lysosomal extracts purified from N27 cells fed with α-syn_f_ (1 μm) and probed for α-syn using antibodies with defined epitopes at 1–50 (*left*) and 91–99 (*right*). Bands migrating at 12 (*) and 8 kDa (**) were detected. *c*, Western blot analysis of lysosomal extracts purified from N27 cells pretreated with α-syn_f_ (1 μm) probed with α-syn antibody (epitope 91–99) before (0 h) and after a 20-h incubation at room temperature. *d*, SDS-PAGE (4–12%) analysis of exogenously added α-syn fibrils (15 μm) to lysosomal extracts from N27 cells (4 and 10 μg) at pH 5 for 20 h and 37 °C. *Asterisk* denotes a 12-kDa band. *e*, LC trace monitored at 210 nm of fibrillar α-syn (15 μm) incubated with 10 μg of lysosomal extracts from N27 cells for 20 h at pH 5 and 37 °C. *mAU*, milli absorbance units. Peptide fragments found in the corresponding peaks by MS analysis are listed in the order of their peptide abundance. All other assigned peaks are shown in Table S5. *Asterisk* denotes assignment of 12-kDa band observed in *panel d.*

To observe α-syn association with lysosomes in treated N27 cells, immunofluorescence experiments using α-syn (epitope 1–50) and CtsL antibodies were performed. After treating N27 cells for 48 h with fibrillar (Fig. 5*a*, *top*; see Fig. S7 for additional fields of view) and soluble α-syn (Fig. 5*a*, *bottom*), immunoreactivity and colocalization were only observed in fibril-treated cells. Images for untreated cells are shown in Fig. S7. After treating with α-syn_f_ (1 μm), α-syn–positive puncta are clearly visible, and most also co-staining with the lysosomal marker CtsL. Monomer treatment showed dispersed immunoreactivity of α-syn and no colocalization with CtsL.

To unequivocally prove that C-terminal truncations are directly derived from lysosomal degradation of α-syn_f_, lysosomes were purified from N27 cells pretreated with exogenous fibrils. A total of 31 T75 flasks containing confluent (∼70%) N27 cells grown for 48 h in the presence of 1 μm fibrils were used to obtain enough lysosomal extract for immunoblotting. Using an N-terminal α-syn antibody (epitope 1–50), full-length α-syn and both 12- and 8-kDa species were observed (Fig. 5*b*). Higher molecular mass bands were also present, indicative of aggregated α-syn. Using an antibody that recognizes an epitope at residues 91–99, only the 12-kDa band could be observed, indicating that the 8-kDa band had lost this epitope region. To ensure that the lysosomal extracts were still active, Western blot analysis was performed after 20 h (Fig. 5*c*). It was evident that proteolysis was still occurring, as the full-length protein was greatly diminished over time, whereas the 12-kDa band, along with some higher molecular mass species, remained, indicating that α-synΔC was more resistant.

Unfortunately, direct MS analysis on the limited amount of lysosomal material from N27 cells was not possible, so we again turned to the use of exogenous α-syn. After adding exogenous α-syn_f_ (15 μm) to lysosomal extracts (2 and 5 μg) from N27 cells, the 12-kDa band, visualized by SDS-PAGE (Fig. 5*d*), was shown by LC-MS to be α-synΔC fragments with or without N-terminal truncations (Fig. 5*e*). At the C-terminal end, the major cleavage site is at Asn-122/Gly-123 along with minor sites at Gln-109/Gly-110 and Gly-114/Asp-115 (Table S5). Cut sites in the N terminus were seen at Phe-4/Met-5, Ser-9/Lys-10, Gly-14/Val-15, and Ala-17/Ala-18, corresponding to fragments 5–122, 10–122, 15–122, and 18–122. These peptides were also observed using fibrils formed *in vitro* from the PD-associated A53T α-syn mutant (Fig. S8; Table S6), indicating that this point mutation does not change fibril degradation at the N and C terminus. These data complement results from lysosomes from *SNCA*^A53T^ mice showing that the 12-kDa species is derived from C-terminal cleavage at Asn-122/Glu-123 and can be attributed to cysteine cathepsin digestion. The fragment 1–114 was also seen from lysosomal activity in *SNCA*^A53T^ mice and is because of CtsB activity; however, the 1–109 fragment appeared to be specific to N27 cells and its origin could not be identified. The N-terminal cut at Gly-14/Val-15 is the consequence of CtsB whereas Ser-9/Lys-10 and Ala-17/Ala-18 result from CtsL activity (27, 29). No assignment of the 8-kDa fragment could be made because of its low abundance. Collectively, these data strongly support that both N- and C-terminal truncations of α-syn originate from incomplete lysosomal degradation and establish that in addition to the 12-kDa species, the 8-kDa band is derived from a fibrillar state of α-syn.

### α-SynΔC generated in situ accelerates α-syn amyloid formation

To assess the aggregation potential of α-syn truncations after incomplete lysosomal digestion, we sought to recapitulate these events using purified cathepsin to investigate its impact on full-length α-syn. Specifically, we asked whether the C-terminal truncations formed *via* limited proteolysis could act as seeds and propagate soluble α-syn aggregation at physiological pH. The cysteine cathepsin AEP was chosen, because this protease has limited substrate specificity on α-syn, cleaving mainly at three positions: Asn-65/Val-66, Asn-103/Glu-104, and Asn-122/Glu-123. Because residues Asn-65 and Val-66 are in the amyloid core, they should be less accessible in the fibrillar state (Fig. 1*b*). Upon fibril digestion, truncations 1–103 and 1–122 should be more abundant. Both of these C-terminal truncations are seen in Lewy bodies (Fig. 1) and have been identified from lysosomal extracts in this work (Figs. 4*c* and 5*e*).

**Figure 6.**
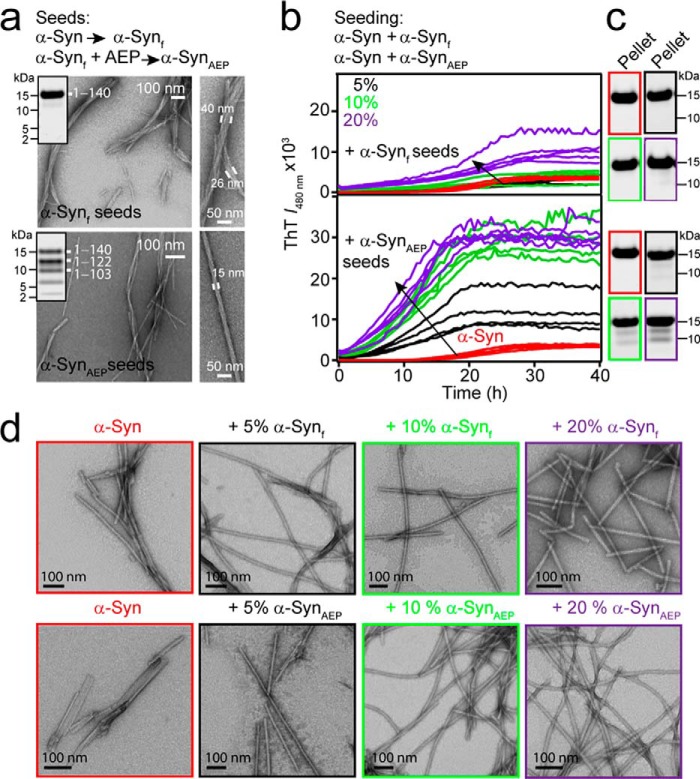
**Effects of C-terminal truncations generated by AEP activity on α-syn amyloid formation.** *a*, scheme of α-syn seed generation in the absence and presence of AEP (200 nm). Representative TEM images of α-syn_f_ in the absence (*top*) and the presence (*bottom*) of AEP. Fibrils with variable widths are shown to be composed of multiple protofibrils (∼5 nm each). *Scale bars* are as shown. *Inset*, SDS-PAGE (4–12%) visualized by Coomassie Blue staining of α-syn_f_ in the absence (*top*) and presence (*bottom*) of AEP. Peptide fragments representing each band was assigned by MS analysis (Table S7). *b*, scheme of α-syn seeding reactions. Aggregation kinetics of α-syn (40 μm) at pH 7.4 in the absence (*red*) and presence of 5 (*black*), 10 (*green*), and 20% (*purple*) fibril seeds alone (α-syn_f_ seeds, *top*) and with AEP digestion (α-syn_AEP_ seeds, *bottom*) and monitored by ThT fluorescence (λ_obs_ = 480 nm). The unseeded data are reproduced in both panels for comparison. Each condition has at least four replicates each. *c*, corresponding SDS-PAGE (4–12%) visualized by Coomassie Blue staining of pelleted samples (100,000 × *g*) taken post aggregation. *d*, representative TEM images taken after 20 h for each condition as indicated. *Scale bar* is 100 nm.

As anticipated, incubation of fibrillar α-syn (100 μm) with AEP (200 nm) at pH 5 for 20 h at 37 °C showed that 1–122 and 1–103 were the dominant species, with a minor population of peptide fragments from cleavage at Asn-65/Val-66 (Fig. 6*a*; Table S7). Attempts to completely remove the C terminus from full-length α-syn_f_ by increasing the AEP was unsuccessful. TEM images of AEP-treated fibrils (α-syn_AEP_) showed multi-protofibril structures (∼5 nm each) that were laterally associated to form fibrils that had a high degree of twisting (Fig. 6*a*, *bottom*). By comparison, greater lateral association of protofibrils was observed for untreated fibrils at pH 5, where some fibrils (>40 nm in diameter) were composed of multiple protofibrils (Fig. 6*a*, *top*).

Next, preformed α-syn_f_ and α-syn_AEP_ (5, 10, and 20%) seeds were incubated with 40 μm α-syn at pH 7.4 with continuous shaking at 37 °C (0–40 h) and monitored by thioflavin T (ThT), an extrinsic fluorophore that increases in intensity upon binding to amyloid fibrils (42). In the absence of seeds, aggregation of soluble α-syn proceeded with a lag phase (∼10 h) followed by a shallow elongation phase (Fig. 6*b*, *red*). Addition of preformed α-syn_f_ seeds had little effect on the observed kinetics, but rather produced higher final ThT signals (Fig. 6*b*, *top*). SDS-PAGE analysis of the pelleted samples taken after 40 h showed comparable protein levels, suggesting the ThT difference is not because of increased fibrillar material (Fig. 6*c*, *top*). In the presence of α-syn_AEP_ seeds (Fig. 6*b*, *bottom*), α-syn aggregation is noticeably faster. The lag phase is hastened as a function of seed concentration, and higher ThT fluorescence signal was reached at the end of the reaction. Again, SDS-PAGE analysis of ultracentrifuged protein samples taken post aggregation suggested that ThT signal changes are not indicative of fibril concentration. Our data clearly show that the truncated fibrils act as superior seeds in promoting α-syn amyloid formation compared with that of the full-length fibrils.

Representative TEM images taken post seeding with α-syn_f_ and α-syn_AEP_ displayed paired protofibrils (5 nm each) laterally associating and forming varying twists (Fig. 6*d*). Because of the heterogenous nature of these fibrils, which also contain some nontwisting fibrils, it is difficult to define morphological differences between seeding conditions. Although the TEM images were inconclusive, the striking differences in ThT signals between α-syn_AEP_–seeded *versus* self-seeded α-syn reactions suggested different fibril polymorphs. To further address this, a second round of seeding was performed. Using the first generation of α-syn_AEP_ seeds (10%), the lag phase was abrogated, and a higher ThT signal was observed compared with the self-seeded reaction (Fig. S9), supporting the propagation of a different polymorph in the α-syn_AEP_–seeded fibrils. These results highlight the potency of the original C-terminal truncated seeds. Although TEM images did not discern obvious morphological differences after a second round of seeding, it is clear that the seeded samples are more homogenous than the nonseeded controls (Fig. S10).

## Discussion

The presence of α-syn truncations in LBs is a feature of PD (Fig. 1*a*), yet the mechanism(s) underlying the formation of these aggregation-prone species is not well-understood. In this work, we could assign >60% of the cleavage sites to lysosomal proteases (Fig. 1*b*), supporting a central role for lysosomal function in PD (43, 44). For the first time, we show that α-synΔC species are found in brain lysosomal preparations from a transgenic *SNCA*^A53T^ PD mouse model. Their abundance was significantly increased in symptomatic *versus* nonsymptomatic mice. Our results are supported by another transgenic *SNCA*^A53T^ mouse study, where immunoblot analysis of mouse brainstem and cortical extracts from symptomatic animals also showed similar increases in full-length and α-syn truncations (45).

We suggest that this enrichment of truncations is not because of a loss of lysosomal proteolysis, as individual cathepsin activities were unchanged (Fig. 3; Fig. S2), but rather, because of a change in the balance between cathepsin and α-syn levels in the lysosome. Under disease conditions, aggregated α-syn is increased in the lysosome. The increased burden leads to incomplete degradation, yielding α-synΔC species (Fig. 2, *f*, *i–l*).

This work revealed that lysosomal fractions in symptomatic *SNCA*^A53T^ mice had α-syn truncations corresponding to 12- and 8-kDa in size (Fig. 2, *j–l*). This was also observed in N27 dopaminergic cells fed with exogenous α-syn fibrils and they originated from truncations of fibrils taking place in the lysosome (Fig. 5). From epitope mapping, likely cleavage sites were defined to be between residues 118 to 123 and 91 to 99, respectively. Unlike the 12-kDa species, which we identified to be the 1–122 segment originating from cysteine cathepsins (Fig. 4*d*), it proved difficult to conclusively identify the 8-kDa fragment from lysosomal degradation of α-syn fibrils. However, through proteolysis of soluble α-syn, LC-MS analysis suggested that it is most likely to be the fragment containing residues 1–90, generated solely by CtsB activity (Fig. S5). This is a newly identified α-syn truncation and further work is needed to establish its importance. Interestingly, the *CTSB* gene encoding CtsB has been recently identified as a PD risk factor (32), up-regulated with increased activity in DLB (33). Moreover, limited proteolysis of α-syn fibrils formed *in vitro* by CtsB have been shown to enhance aggregation of endogenous α-syn in cells (31), providing evidence for a potential role for CtsB in PD pathogenesis.

In addition to C-terminal truncations, four N-terminal truncations (Phe-4/Met-5, Met-5/Lys-6, Ser-9/Lys-10, and Gly-14/Val-15) were observed in both *SNCA*^A53T^ and N27 samples (Figs. 4*c* and 5*e*). Only the N-terminal truncation at residue 5 is found in LBs; thus, the relevance of the other sites remains to be defined. Because it has been shown that removal of 10 and 30 residues in the N terminus modulates fibril formation (15), these truncations will also likely influence α-syn aggregation. Hence, the interplay of N- and C-terminal truncations (*e.g.* residues 10–122) in the mechanism of α-syn amyloid assembly warrants further investigation.

A pathological implication for α-synΔC species was clearly demonstrated by the seeding potency of C-terminal truncations 1–103 and 1–122 generated via AEP digestion of preformed fibrils (Fig. 6*b*). We propose that under disease conditions, the burden of α-syn accumulation and aggregation overwhelms lysosomal degradation, leading to incomplete proteolysis of fibrils and the buildup of α-synΔC species. If released from the lysosome, α-synΔC species would recruit and propagate aggregation of cytosolic α-syn. This proposed mechanism is supported by observations of lysosomal rupture induced by α-syn (46–48). It has been shown that α-syn aggregates colocalize with galectin-3, a marker of endolysosomal membrane rupture (47), although the molecular mechanisms causing lysosomal rupture remain to be elucidated. Of note, *in vivo* studies have also suggested that intercellular transmission of α-syn aggregates occurs via the endolysosome pathway (38, 39). One study proposes α-syn fibrils traffic inside lysosomes in tunneling nanotubes, contributing to intercellular transfer of α-syn fibrils (49). Taken together, these events substantiate the connection between lysosomes and amyloid propagation in PD.

With these identified α-synΔC species, quantification of their physiological concentrations would be of particular interest, as some are readily found in healthy tissue (50). One intriguing implication is how they could play a role in the reciprocal relationship between levels of α-syn and the lysosomal hydrolase glucocerebrosidase (GCase) (51), which is currently recognized as the most prevalent genetic risk factor for the development of PD and DLB (52, 53). Although the specific role of GCase activity in modulating α-syn levels remains controversial, studies have shown that enhancing GCase activity is a potential therapeutic strategy for synucleinopathies (54–57). Interestingly, reduced levels of GCase are reported to facilitate cell-to-cell transmission of α-syn (58). Because GCase binds to the C-terminal region of α-syn, from residues 118 to 137 (59–61), the physical interaction between full-length α-syn and GCase would inhibit C-terminal truncation via steric hindrance, which would have a protective effect in reducing the presence of potent seeds for amyloid propagation. On the other hand, once truncations such as those observed in this study are formed (1–122, 1–114, 1–103, and 1–90) as well as others found in LBs (1–119, 1–115, and 1–101), this association would be mitigated. Further work is needed to establish the importance of this physical interaction in the context of PD where α-syn truncations are enriched.

Based on our results, a targeted therapeutic strategy is to decrease the production of α-synΔC species in lysosomes. Promising results from deletion of AEP were recently reported to reduce aggregation and dopaminergic cell death in mice overexpressing human α-syn (28). Importantly, we have previously shown that with sufficient amounts of CtsL, this cysteine cathepsin is fully capable of cannibalizing α-syn fibrils (27, 29). However, from our studies in *SNCA*^A53T^ mouse brain samples, it is clear that there is limited CtsL activity in lysosomes. Thus, one therapeutic approach might be to ameliorate amyloid load by enhancing CtsL activity in the lysosome.

## Experimental procedures

### Proteins and reagents

N-terminally acetylated wildtype and A53T α-syn were expressed in BL21(DE3) using human α-syn (pRK172) (62) and yeast NatB genes (63) and purified as described previously (64). N-terminally acetylated α-syn was used because it is the ubiquitous, native form *in vivo* (2). Filtered buffers (0.22 μm) were used for all experiments. Purity of α-syn was assessed by SDS-PAGE (NuPAGE 4–12% Bis-Tris, Invitrogen) and confirmed by MS (NHLBI Biochemistry Core). Purified protein was stored at −80 °C until use. Protein concentrations were determined using a molar extinction coefficient estimated on basis of amino acid content: ϵ_280 nm_ = 5120 m^−1^ cm^−1^. N27 rat dopaminergic neuronal cell line was purchased from EMD Millipore (catalog no. SCC048). Recombinant human AEP was purchased from R&D Systems (catalog no. 2199-CY-010). Primary α-synuclein antibodies used were mouse monoclonal (Syn-1, BD Biosciences), rabbit monoclonal EP1646Y (ab51252, Abcam), rabbit monoclonal (ab138501, Abcam), mouse monoclonal (ab27766 (LB509), Abcam), rabbit polyclonal (ab6176, Abcam), rabbit polyclonal (ab53726, Abcam), mouse monoclonal (ab80627 (Syn211), Abcam), rabbit polyclonal (ab52168, Abcam), sheep polyclonal (ab21976, Abcam), and rabbit polyclonal Ser-129–phosphorylated α-synuclein (ab59264, Abcam). Primary rabbit monoclonal CtsL (ab6314, Abcam) was used. HRP-conjugated goat anti-rabbit, anti-mouse, and anti-sheep secondary antibodies were purchased from Kirkegaard & Perry Laboratories, Inc. (Gaithersburg, MD). Alexa Fluor 488 conjugated donkey polyclonal anti-rabbit (ab150073, Abcam) and Alexa Fluor 532 goat anti-mouse (A-11002, Thermo Fisher Scientific) secondary antibodies were used for immunofluorescence imaging. Other reagents were purchased and used as received: Thioflavin T (T3516–5G, Sigma), DTT (catalog no. 194821, MP Biomedicals), PePA (P5318–5MG, Sigma-Aldrich), CA-074 (205530–1MG, Calbiochem), z-FY(*t*-Bu)-DMK (219427–5MG, Millipore Sigma), AENK (533796, Millipore Sigma), LeuP (L2884.5MG, Sigma-Aldrich), z-RR-AMC (catalog no. 219392, Calbiochem), z-FR-AMC (catalog no. 03–32-1501–25MG, Calbiochem), z-AAN-AMC (catalog no. 4033201, Bachem), and MCA-GKPILEFRKL(Dnp)-D-R-NH_2_ (catalog no. 219360–1MG, Calbiochem).

### Lysosome isolation from wt, ∼SNCA^A53T^, and SNCA^A53T^ mice

Mice with a human *SNCA*^A53T^ transgene (34) were housed and bred under NHGRI Animal Care and Use Committee–approved protocols. Mice were euthanized when progressive neurodegenerative symptoms develop, typically at ∼16 months of age. Symptoms were identified by clinical deterioration beginning with weight loss and progressing to abnormal gait and posture. When they become unable to ambulate, we euthanize them. Age-matched nonsymptomatic *SNCA*^A53T^ littermates as well as nontransgenic mice were euthanized in parallel. A total of six brains, two from each group were used for lysosome isolation. Samples were kept on ice at all times. Brains were gently homogenized using a Dounce homogenizer (∼50 strokes) and spun at 500 × *g*, and soluble lysates were combined with OptiPrep (lysosomal enrichment kit, Pierce) to a final concentration of 15% and placed on top of a discontinuous density gradient with the following steps from top to bottom: 17, 20, 23, 27, and 30%. After centrifugation for 2 h at 145,000 × *g*, the top fraction containing the lysosomes was collected. The lysosomal fraction was diluted at least three times with PBS and pelleted by centrifugation for 30 min at 16,100 × *g*. The pellets were washed once with PBS and then resuspended in 50 mm sodium acetate (NaOAc), 20 mm NaCl, pH 5 buffer and centrifuged again at 16,100 × *g* for 30 min. Pellets were stored at −80 °C. To generate lysosomal extracts, purified lysosomes were frozen-thawed four times. Total protein concentrations in the resulting lysosome lysates were determined using the Bradford assay (detergent compatible kit from Pierce Biotechnology): For biological replicate 1: *wt* (0.54 mg/ml), ∼*SNCA*^A53T^ (0.75 mg/ml), and *SNCA*^A53T^ (0.5 mg/ml) and biological replicate 2: *wt* (1.53 mg/ml), ∼*SNCA*^A53T^ (0.9 mg/ml) and *SNCA*^A53T^ (0.58 mg/ml).

### Gel electrophoresis and immunoblotting analysis of lysosomes

For Western blot analysis, lysosomal extracts (1–5 μg total protein) were separated by SDS-PAGE (NuPAGE 4–12% Bis-Tris, Invitrogen) and stained with Coomassie SimplyBlue Safe Stain (Invitrogen) or Ponceau S or transferred to PVDF (0.45 μm) membranes for Western blot analysis. Transfer conditions using the XCell II Blot Module (Invitrogen, EI9051) with a constant 25 V, 125 mA for 90 min. Posttransfer PVDF membranes were fixed for 30 min with 4% paraformaldehyde in PBS at room temperature. Blots were blocked in Tris-buffered saline containing 0.1% Tween 20 (Sigma) and 5% nonfat dry milk (170–6404, Bio-Rad) for 1 h at room temperature. The blocked membrane was incubated in blocking buffer containing the following v/v of primary antibodies: Syn-1 (1:1000), EP1646Y (1:1000), ab138501 (1:2000), LB509 (1:5000), ab6176 (1:1000), ab53726 (1:1000), Syn211 (1:2000), ab52168 (1:1000), ab21976 (1:5000), and ab59264 (1:5000) overnight at 4 °C, followed by four 10-min washes. Membranes were incubated in blocking buffer containing HRP-conjugated secondary antibody (1:10,000) for 1 h at room temperature. Blots were developed using ECL chemiluminescent substrate (Thermo Fisher). Film (876 1520, Kodak) was exposed for varying times between 10 s to 5 min. All blots were performed at least two times for each biological replicate.

### Cathepsin activity assays

Cathepsin activity was measured using substrates (Ac-RR-AMC for CtsB, MCA-GKPILEFRKL(Dnp)-D-R-NH_2_ for CtsD, Ac-FR-AMC for CtsL, and z-AAN-AMC) at a final concentration of 200 μm (for CtsB, CtsL, and AEP) and 20 μm (for CtsD) with 1 μg total protein lysosomal extract in pH 5 buffer. All buffers contained 5 mm DTT. Reactions were performed in polypropylene 384-well flat-bottom microplates (Greiner Bio-One) containing 50-μl solution incubated at 37 °C using a microplate reader (Tecan Infinite M200 Pro). For each biological replicate set, a total of three repetitions were performed. CtsB, CtsL, and AEP (excitation and emission wavelengths at 360 and 460 nm, respectively) and CtsD fluorescence (excitation and emission wavelengths at 328 and 393 nm, respectively) were recorded as a function of time (0–90 min).

### Fibril formation

To prepare fibrils, α-syn was exchanged into pH 5 buffer (50 mm NaOAc, 20 mm NaCl) using a PD-10 column (GE Healthcare) and filtered through YM-100 filters (Millipore) to remove any preformed aggregates prior to aggregation. Aggregation was performed in microcentrifuge tubes (1.5-ml Protein LoBind tubes, catalog no. 022431081, Eppendorf) containing 1 ml solution (α-syn = 100 μm) with continuous shaking at 600 rpm at 37 °C for 3 days in a Mini-Micro 980140 shaker (VWR).

### Degradation reactions of recombinant α-syn

In microcentrifuge tubes (1.5-ml Protein LoBind tubes, catalog no. 022431081, Eppendorf), soluble α-syn and preformed α-syn fibrils (15 and 30 μm) were incubated with lysosomal extracts. The amounts of lysosomal extracts were 1–5 and 2–30 μg for soluble and fibrillar α-syn, respectively. For inhibition experiments, lysosomal extracts were preincubated with 1 μm of each inhibitor for 30 min at room temperature prior to the addition of α-syn. Limited proteolysis of α-syn_f_ (100 μm) was performed using 200 nm of AEP. All reactions were carried out in a total volume of 100 μl reaction buffer (50 mm NaOAc, 20 mm NaCl, 5 mm DTT, pH 5), agitated at 600 rpm at 37 °C for 20 h in a Mini-Micro 980140 shaker (VWR).

### LC-MS

Proteolyzed samples (5–20 μl) were separated using an HPLC (Agilent 1100 series, Agilent Technologies) on a reverse phase C18 column (Zorbax, 2.1 × 50 mm, 3.5 μm, Agilent Technologies) and introduced into the mass spectrometer as described (65, 66). Fibrillar samples were preincubated to a final concentration of 2 m guanidium hydrochloride before injection. Positive ion electrospray ionization (ESI) mass spectra for intact protein (peptides) of α-syn were obtained with an Agilent G1956B mass selective detector (MSD) equipped with an ESI interface (Agilent Technologies). The HPLC systems and MSD were controlled and data were analyzed using LC/MSD ChemStation software (Rev. B.04.03, Agilent Technologies).

### Live cell imaging of N27 dopaminergic cells

N27 dopaminergic cells were maintained in RPMI media containing 10% fetal bovine serum and supplemented with 2% penicillin/streptomycin at 37 °C in 5% CO_2_ atmosphere. N27 cells at 70–80% confluency were trypsinized by the addition of 0.05% trypsin-EDTA for 5 min. Resuspended cells (50–80 μl) and fresh media (300 μl) were added to Labtek 8-well chambers precoated with poly-l-lysine (P8920, Sigma). Exogenous soluble and fibrillar α-syn in pH 5 buffer was diluted with media to desired final concentrations (100 nm and 1 μm) and added prior to imaging. Samples were imaged using a 20× air objective on a Zeiss 780 confocal microscope (NHLBI Light Microscopy Core) and maintained at 37 °C in CO_2_ atmosphere.

### Immunofluorescence analysis of N27 cells

N27 cells were plated on no. 1.5 glass coverslips in 6-well plates and allowed to recover for 24 h. Cells were then incubated for 48 h at 37 °C in fresh media containing the desired concentration of soluble (exchanged into pH 5 buffer) or fibrillar (preformed in pH 5 buffer, bath sonicated 15 min before use) α-syn. Cells were fixed with 2% paraformaldehyde in PBS buffer for 15 min and washed with PBS. Cells were then permeabilized with 0.5% Triton X-100 and 3% BSA in PBS for 7 min and blocked with 0.2% Triton X-100 and 3% BSA in PBS for 1 h. Finally, cells were stained for 1 h with primary antibody (mouse CtsL (ab6314) and rabbit α-syn (ab51252), diluted 1:50–1:100 in blocking buffer), washed with blocking buffer, stained for 1 h with secondary antibody (goat α-mouse Alexa Fluor 532 (A-11002) and donkey α-rabbit Alexa Fluor 488 (ab150073), diluted 1:1000 in blocking buffer), and washed with blocking buffer. All procedures were performed at room temperature. Coverslips were stored in PBS at 4 °C in the dark before imaging. Samples were imaged using a UPLSAPO 100×/1.35 NA silicon oil objective (Olympus) on an OlympusIX73 inverted microscope fitted with a Thorlab Confocal Laser Scanner (CLS-SL) fiber coupled to a multichannel CMLS-E laser source. Alexa Fluor 488 and 532 emission were excited at 488 and 532 nm and collected using 512 ± 25 and 582 ± 75 nm band-pass filters, respectively, by two independent high-sensitivity GaAsP photomultiplier tubes. A 45-μm pinhole was used and the scale was 0.1 μm/pixel. Two independent treatments were imaged and analyzed. Images were analyzed with Fiji (67).

### Lysosome isolation from rat N27 dopaminergic cells

N27 cells from 31 T75 flasks containing 10 ml of cell culture at 70–80% confluency was scraped using a cell scraper and centrifuged at 3220 × *g* for 10 min. Cell pellet was resuspended in PBS and gently homogenized using a Dounce homogenizer (200 strokes). Lysosome purification proceeded as described for mice lysosome isolation. The protein concentration in the resulting lysates was determined to be 0.52 mg/ml using the Bradford assay (detergent compatible kit from Pierce Biotechnology).

### Aggregation kinetics

Aggregation was performed in sealed black polypropylene 384-well flat-bottom microplates (781209, Greiner Bio-One) in the absence or presence of preformed α-syn_f_ seeds and continuously shaken linearly (1.0 mm, 173.9 rpm) at 37 °C using a microplate reader (Tecan Infinite M200 Pro). Seeds were added (2.5, 5, and 10 μl from aggregation or digestion reactions performed at 100 μm as described above) to a solution (α-syn = 50 μm in 20 mm sodium phosphate and 100 mm NaCl, pH 7.4) to a final volume of 40 μl. Each well also was supplemented with a 2-mm glass bead. The final α-syn concentration is 40 μm. ThT (10 μm) fluorescence (excited and monitored at 415 and 480 nm, respectively) was recorded as a function of time. A total of three independent experiments was performed with at least four replicates on each plate.

### TEM

Samples (10 μl) were put on TEM grids (400-mesh Formvar and carbon-coated copper, Electron Microscopy Sciences) for approximately 2 min and wicked away by filter paper. Addition of 10 μl of deionized water was then applied and wicked away immediately. A solution of 1% uranyl acetate (10 μl) is placed on the grid for 2 min, wicked away, and air-dried. TEM was performed using a JEOL JEM 1200EX transmission electron microscope (accelerating voltage 80 keV) equipped with an AMT XR-60 digital camera (NHLBI EM Core Facility).

## Author contributions

R. P. M. and J. C. L. conceptualization; R. P. M., S. M. L., and K. E. H. data curation; R. P. M. formal analysis; R. P. M. writing-original draft; N. T. and E. S. resources; E. S. and J. C. L. writing-review and editing; J. C. L. supervision.

## Supplementary Material

Supporting Information

## Footnotes

2 The abbreviations used are:

PD: Parkinson's disease
LBs: Lewy bodies
α-syn: α-synuclein
Cts: cathepsin
ΔC: C-terminal truncations
AEP: asparagine endopeptidase
DLB: dementia with LBs
PePA: pepstatin A
TEM: transmission electron microscopy
ThT: thioflavin T
GCase: glucocerebrosidase
ESI: electrospray ionization
MSD: mass selective detector.
