# Supplementary material for: C-terminal α-synuclein truncations are linked to cysteine cathepsin activity in Parkinson's disease
Source: J Biol Chem. 2019 May 15;294(25):9973–84. doi: 10.1074/jbc.RA119.008930 (PMC6597809; doi:10.1074/jbc.RA119.008930)
Supplement: Supporting Information [file supp_294_25_9973__index.html]

C-terminal α-synuclein truncations are linked to cysteine cathepsin activity in Parkinson's disease — C-terminal α-syn truncations — Supporting Information 

# C-terminal α-synuclein truncations are linked to cysteine cathepsin activity in Parkinson's disease

## Supporting Information

- Supporting Information (to be published online) - Supporting Information
