# Supplementary material for: C-terminal α-synuclein truncations are linked to cysteine cathepsin activity in Parkinson's disease
Source: J Biol Chem. 2019 May 15;294(25):9973–84. doi: 10.1074/jbc.RA119.008930 (PMC6597809; doi:10.1074/jbc.RA119.008930)
Supplement: Supporting Information [file supp_RA119.008930_144969_2_supp_331443_prjzt4.pdf]

*Supporting Information for:*

# Linking C-terminal α-Synuclein Truncations to Cysteine Cathepsin Activity in Parkinson's Disease

*Ryan P. McGlinchey[1], Shannon M. Lacy[1], Katherine E. Huffer[1], Nahid Tayebi[2], Ellen Sidransky[2] and Jennifer C. Lee[1,*]*

[1]Laboratory of Protein Conformation and Dynamics, Biochemistry and Biophysics Center, National Heart, Lung, and Blood Institute and [2]Medical Genetics Branch, National Human Genome Research Institute, National Institutes of Health, Bethesda, Maryland 20892

*E-mail: leej4@nhlbi.nih.gov, Tel: 301-827-0723

Contents:

- Table S1: Mass spectrometry data for degradation of α-syn fibrils using *SNCA*$^{A53T}$ lysosomes
- Table S2: Mass spectrometry data for degradation of soluble α-syn using *SNCA*$^{A53T}$ lysosomes
- Table S3: Mass spectrometry data of α-syn fibril degradation using selective cathepsin inhibitors
- Table S4: Mass spectrometry data of soluble α-syn degradation using selective cathepsin inhibitors
- Table S5: Mass spectrometry data for degradation of α-syn fibrils using N27 lysosomes
- Table S6: Mass spectrometry data for degradation of A53T fibrils using N27 lysosomes
- Table S7: Mass spectrometry data for asparagine endopeptidase limited proteolysis
- Figure S1: Sequence alignment of human and mouse α-synuclein
- Figure S2: Cathepsin activities in lysosomes from *SNCA*$^{A53T}$mice
- Figure S3: TEM images α-syn fibrils formed in pH 5 buffer
- Figure S4: Selective inhibitor experiments of soluble α-syn incubated with lysosomes from *SNCA*$^{A53T}$ mice

- Figure S5: SDS-PAGE analysis of α-syn fibril degradation using selective cathepsin inhibitors
- Figure S6: Exogenous addition of α-syn to N27 rat dopaminergic neuronal cells
- Figure S7: Immunofluorescence images of N27 rat dopaminergic cells co-stained with α-syn and CtsL
- Figure S8: SDS-PAGE analysis for degradation of α-syn and A53T fibrils using N27 lysosomes
- Figure S9: Aggregation kinetics of seeded α-syn from asparagine endopeptidase treatment
- Figure S10: TEM images of α-syn fibrils formed from a second round of seeding with asparagine endopeptidase treatment

**Table S1.** MS analysis of N-terminally acetylated α-syn$_f$ (15 and 30 µM) incubated with lysosomes from *SNCA*$^{A53T}$ mice (5 µg total protein) for 20 h at pH 5 and 37 °C.

| Observed Mass (Da) | Theoretical Mass (Da) | Position in α-syn sequence |
|---|---|---|
| 14501.45 | 14502.16 | 1–140 |
| 14430.42 | 14431.08 | 1–139 |
| 13966.79 | 13967.57 | 5–140 |
| 13895.70 | 13896.49 | 5–139 |
| 13835.82 | 13836.37 | 6–140 |
| 13765.54 | 13765.29 | 6–139 |
| 13451.01 | 13450.91 | 10–140 |
| 13379.15 | 13379.83 | 10–139 |
| 12936.98 | 12937.32 | 15–140 |
| 12864.91 | 12866.24 | 15–139 |
| 12583.00 | 12583.20 | 1–124 |
| 12382.18 | 12383.00 | 1–122 |
| 12047.96 | 12048.61 | 5–124 |
| 11847.80 | 11848.41 | 5–122 |
| 11498.53 | 11499.14 | 1–114 |
| 11331.81 | 11331.76 | 10–122 |
| 10964.63 | 10964.48 | 5–114 |
| 10817.57 | 10818.16 | 15–122 |
| 9934.47 | 9934.23 | 15–114 |

**Table S2.** MS analysis of soluble N-terminally acetylated α-syn (15 and 30 μM) incubated with lysosomes from *SNCA*$^{A53T}$ mice (1, 2.5, and 5 μg total protein) for 20 h at pH 5 and 37 °C.

| Observed Mass (Da) | Theoretical Mass (Da) | Position in α-syn sequence |
|---|---|---|
| 14501.48 | 14502.16 | 1–140 |
| 14430.47 | 14431.08 | 1–139 |
| 13966.83 | 13967.57 | 5–140 |
| 13895.69 | 13896.49 | 5–139 |
| 12936.91 | 12937.32 | 15–140 |
| 12865.53 | 12866.24 | 15–139 |
| 12736.71 | 12737.12 | 15–138 |
| 12582.43 | 12583.20 | 1–124 |
| 12047.82 | 12048.61 | 5–124 |
| 10345.90 | 10345.86 | 1–103 |
| 8957.74 | 8958.26 | 1–90 |
| 8887.03 | 8887.18 | 1–89 |
| 8780.77 | 8781.02 | 15–103 |
| 8423.30 | 8423.67 | 5–90 |
| 7870.75 | 7871.51 | 65–140 |
| 7403.01 | 7403.50 | 1–73 |
| 7392.84 | 7393.42 | 15–90 |
| 6448.22 | 6448.42 | 1–62 |
| 6190.89 | 6191.17 | 1–60 |
| 5490.30 | 5490.83 | 91–139 |
| 5264.16 | 5263.08 | 1–51 |
| 5205.79 | 5206.04 | 1–50 |
| 5185.01 | 5185.50 | 95–140 |
| 5113.95 | 5114.42 | 95–139 |
| 5055.36 | 5055.72 | 39–90 |
| 4002.83 | 4003.33 | 95–130 |
| 3708.11 | 3708.27 | 1–36 |
| 3521.91 | 3522.10 | 1–34 |

**Table S3.** MS analysis of N-terminally acetylated α-syn$_f$ (15 and 30 µM) incubated with lysosomes from *SNCA*$^{A53T}$ mice (5 µg total protein) in the presence of selective protease inhibitors for 20 h at pH 5 and 37 °C.

*SNCA*$^{A53T}$ lysosomes (5 µg) + α-syn$_f$ (15 and 30 µM) + PepA

| Observed Mass (Da) | Theoretical Mass (Da) | Position in α-syn sequence |
|---|---|---|
| 14501.23 | 14502.16 | 1–140 |
| 13835.45 | 13836.37 | 6–140 |
| 13449.98 | 13450.91 | 10–140 |
| 12936.18 | 12937.32 | 15–140 |
| 12381.93 | 12383.00 | 1–122 |

*SNCA*$^{A53T}$ lysosomes (5 µg) + α-syn$_f$ (15 and 30 µM) + CA-074

| Observed Mass (Da) | Theoretical Mass (Da) | Position in α-syn sequence |
|---|---|---|
| 14501.98 | 14502.16 | 1–140 |
| 14429.80 | 14431.08 | 1–139 |
| 13966.04 | 13967.57 | 5–140 |
| 13895.22 | 13896.49 | 5–139 |
| 12582.99 | 12583.20 | 1–124 |
| 12382.73 | 12383.00 | 1–122 |

*SNCA*$^{A53T}$ lysosomes (5 µg) + α-syn$_f$ (15 and 30 µM) + z-FY-DMK

| Observed Mass (Da) | Theoretical Mass (Da) | Position in α-syn sequence |
|---|---|---|
| 14502.01 | 14502.16 | 1–140 |
| 14430.93 | 14431.08 | 1–139 |
| 13967.40 | 13967.57 | 5–140 |
| 13896.55 | 13896.49 | 5–139 |
| 13836.54 | 13836.37 | 6–140 |
| 13451.01 | 13450.91 | 10–140 |
| 12937.76 | 12937.32 | 15–140 |
| 12866.10 | 12866.24 | 15–139 |
| 12583.00 | 12583.20 | 1–124 |
| 12382.67 | 12383.00 | 1–122 |
| 12047.96 | 12048.61 | 5–124 |
| 11497.97 | 11499.14 | 1–114 |
| 11848.22 | 11848.41 | 5–122 |
| 10964.63 | 10964.48 | 5–114 |
| 10818.52 | 10818.16 | 15–122 |
| 9934.47 | 9934.23 | 15–114 |

**Table S4.** MS analysis of soluble N-terminally acetylated α-syn (15 μM) incubated with lysosomes from *SNCA*$^{A53T}$ mice (2.5 μg total protein) in the presence of selective protease inhibitors for 20 h at pH 5 and 37 °C.

*SNCA*$^{A53T}$ lysosomes (2.5 μg)
+ α-syn (15 μM) + PepA

| Observed Mass (Da) | Theoretical Mass (Da) | Position in α-syn sequence |
|---|---|---|
| 14501.05 | 14502.16 | 1–140 |
| 14429.96 | 14431.08 | 1–139 |
| 12936.24 | 12937.32 | 15–140 |
| 12865.16 | 12866.24 | 15–139 |
| 8957.43 | 8958.26 | 1–90 |
| 7403.01 | 7403.50 | 1–73 |
| 7392.87 | 7393.42 | 15–90 |
| 6762.10 | 6762.76 | 1–65 |
| 6447.91 | 6448.42 | 1–62 |
| 6190.76 | 6191.17 | 1–60 |
| 5561.61 | 5561.91 | 91–140 |
| 5490.09 | 5490.83 | 91–139 |
| 5262.52 | 5263.08 | 1–51 |
| 3708.02 | 3708.27 | 1–36 |
| 3521.77 | 3522.10 | 1–34 |

*SNCA*$^{A53T}$ lysosomes (2.5 μg)
+ α-syn (15 μM) + z-FY-DMK

| Observed Mass (Da) | Theoretical Mass (Da) | Position of α-syn sequence |
|---|---|---|
| 14500.96 | 14502.16 | 1–140 |
| 13966.13 | 13967.57 | 5–140 |
| 12936.11 | 12937.32 | 15–140 |
| 8957.43 | 8958.26 | 1–90 |
| 8422.80 | 8423.6 | 5–90 |
| 7870.75 | 7871.5 | 65–140 |
| 6762.13 | 6762.76 | 1–65 |
| 6447.85 | 6448.42 | 1–62 |
| 6190.73 | 6191.17 | 1–60 |
| 5562.30 | 5561.91 | 91–140 |
| 5262.41 | 5263.08 | 1–51 |
| 5184.78 | 5185.50 | 95–140 |
| 5113.66 | 5114.42 | 95–139 |
| 5055.11 | 5055.72 | 39–90 |
| 3707.93 | 3708.27 | 1–36 |
| 3521.72 | 3522.10 | 1–34 |

*SNCA*$^{A53T}$ lysosomes (2.5 μg)
+ α-syn (15 μM) + CA-074

| Observed Mass (Da) | Theoretical Mass (Da) | Position in α-syn sequence |
|---|---|---|
| 14500.98 | 14502.16 | 1–140 |
| 14429.80 | 14431.08 | 1–139 |
| 13966.04 | 13967.57 | 5–140 |
| 13895.22 | 13896.49 | 5–139 |

*SNCA*$^{A53T}$ lysosomes (2.5 μg)
+ α-syn (15 μM) + AENK

| Observed Mass (Da) | Theoretical Mass (Da) | Position of α-syn sequence |
|---|---|---|
| 14500.88 | 14502.16 | 1–140 |
| 13966.10 | 13967.57 | 5–140 |
| 13895.04 | 13896.49 | 5–139 |
| 12936.12 | 12937.32 | 15–140 |
| 12865.03 | 12866.24 | 15–139 |
| 10598.71 | 10599.61 | 39–140 |
| 8957.37 | 8958.26 | 1–90 |
| 8422.80 | 8423.6 | 5–90 |
| 7392.87 | 7393.42 | 15–90 |
| 6447.85 | 6448.42 | 1–62 |
| 6190.73 | 6191.17 | 1–60 |
| 5561.61 | 5561.91 | 91–140 |
| 5184.76 | 5185.50 | 95–140 |
| 5113.63 | 5114.42 | 95–139 |
| 3707.92 | 3708.27 | 1–36 |
| 3521.75 | 3522.10 | 1–34 |

**Table S5.** MS analysis of N-terminally acetylated α-syn$_f$ (15 µM) incubated with lysosomes from N27 cells (2 and 5 µg total protein) for 20 h at pH 5 and 37 °C.

| Observed Mass (Da) | Theoretical Mass (Da) | Position of α-syn sequence |
|---|---|---|
| 14502.75 | 14502.16 | 1–140 |
| 12383.56 | 12383.00 | 1–122 |
| 11849.75 | 11848.41 | 5–122 |
| 11499.14 | 11499.14 | 1–114 |
| 11332.14 | 11331.76 | 10–122 |
| 10957.43 | 10957.47 | 1–109 |
| 10818.64 | 10818.64 | 15–122 |
| 10549.01 | 10548.82 | 18–122 |

**Table S6.** MS analysis of N-terminally acetylated A53T$_f$ (15 µM) incubated with lysosomes from N27 cells (2 and 5 µg total protein) for 20 h at pH 5 and 37 °C.

A53T$_f$ (15 µM) + N27 lysosomes (5 µg)

| Observed Mass (Da) | Theoretical Mass (Da) | Position of α-syn sequence |
|---|---|---|
| 14532.52 | 14532.18 | 1–140 |
| 13482.05 | 13480.94 | 10–140 |
| 12413.51 | 12413.03 | 1–122 |
| 11848.68 | 11848.41 | 5–122 |
| 11529.40 | 11529.10 | 1–114 |
| 11362.00 | 11361.78 | 10–122 |
| 10987.85 | 10987.50 | 1–109 |
| 10848.48 | 10848.19 | 15–122 |
| 10578.91 | 10578.84 | 18–122 |
| 9936.62 | 9936.25 | 10–109 |
| 9695.04 | 9694.92 | 18–114 |
| 9153.69 | 9153.31 | 18–109 |

**Table S7.** MS analysis of N-terminally acetylated α-syn$_f$ (100 µM) incubated with purified AEP (200 nM) for 20 h at pH 5 and 37 ºC.

| α-syn$_f$ (100 µM) + human AEP (200 nM) | | |
|---|---|---|
| Observed Mass (Da) | Theoretical Mass (Da) | Position of α-syn sequence |
| 14501.16 | 14502.16 | 1–140 |
| 10345.01 | 10345.86 | 1–103 |
| 12382.41 | 12383.00 | 1–122 |
| 12093.71 | 12094.72 | 3–122 |
| 11325.06 | 11325.88 | 3–115 |
| 10057.01 | 10057.58 | 3–103 |
| 6762.35 | 6762.66 | 1–65 |
| 6474.00 | 6474.48 | 3–65 |
| 3600.80 | 3601.12 | 66–103 |
| 2136.69 | 2137.17 | 123–140 |
| 2054.03 | 2055.15 | 104–122 |
| 1285.91 | 1286.31 | 104–115 |
| 607.27 | 607.62 | 136–140 |

Human α-syn MDVFMKGLSKAKEGVVAAAEKTKQGVAEAAGKTKEGVLYVGSKTKEGVVHGVATVAEKTK$_{60}$
mouse α-syn MDVFMKGLSKAKEGVVAAAEKTKQGVAEAAGKTKEGVLYVGSKTKEGVVHGVTTVAEKTK$_{60}$

human α-syn EQVTNVGGAVVTGVTAVAQKTVEGAGSIAAATGFVKKDQLGKNEEGAPQEGILEDMPVDP$_{120}$
mouse α-syn EQVTNVGGAVVTGVTAVAQKTVEGAGNIAAATGFVKKDQMGKGEEGYPQEGILEDMPVDP$_{120}$

human α-syn DNEAYEMPSEEGYQDYEPEA$_{140}$
mouse α-syn GSEAYEMPSEEGYQDYEPEA$_{140}$

**Figure S1.** Sequence alignment of human and mouse α-synuclein. Differences in amino acids of the human sequence are colored red.

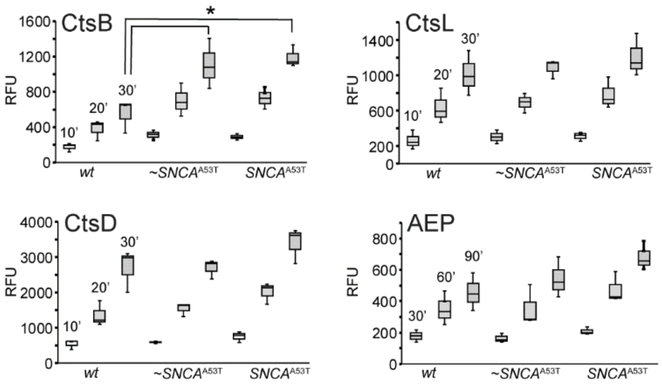


**Figure S2.** Endogenous protease activities in lysosomes from *wt*, non- (~*SNCA*$^{A53T}$), and symptomatic (*SNCA*$^{A53T}$) mice measured by fluorogenic substrates. Ac-RR-AMC for CtsB, Ac-FR-AMC for CtsL, MCA-GKPILEFRKL(Dnp)-D-R-$NH_2$ for CtsD, and AENK-AMC for AEP were incubated with brain lysosomal extracts (1 µg total protein) at pH 5 with 5 mM DTT and 37 °C. Fluorescence was recorded as a function of time (10 to 90 min) and relative fluorescence units (RFU) are reported ($n = 3$). CtsB activity of *wt* versus pre-symptomatic and *wt* versus symptomatic lysosome have statistical significance (*t*-test) *$p$<0.05.

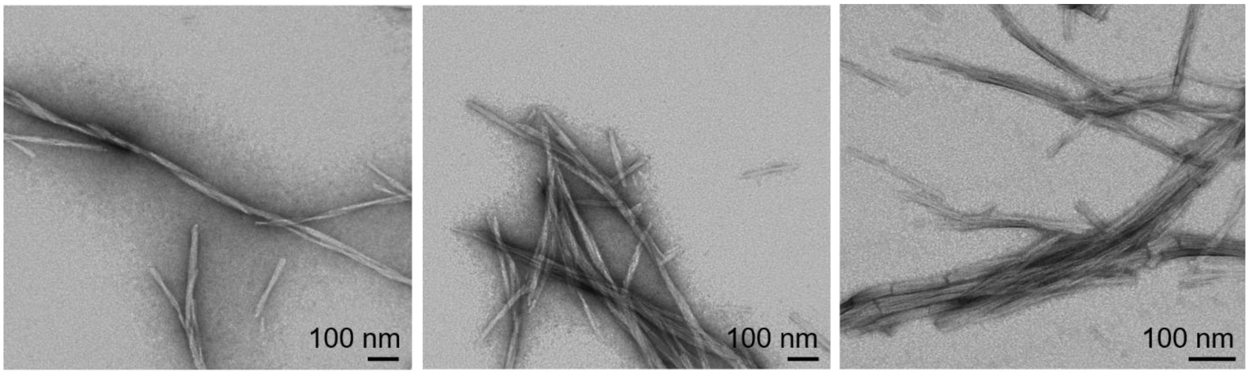


**Figure S3.** Representative TEM images of α-syn$_f$ (15 μM) formed in pH 5 buffer. Scale bars are as shown.

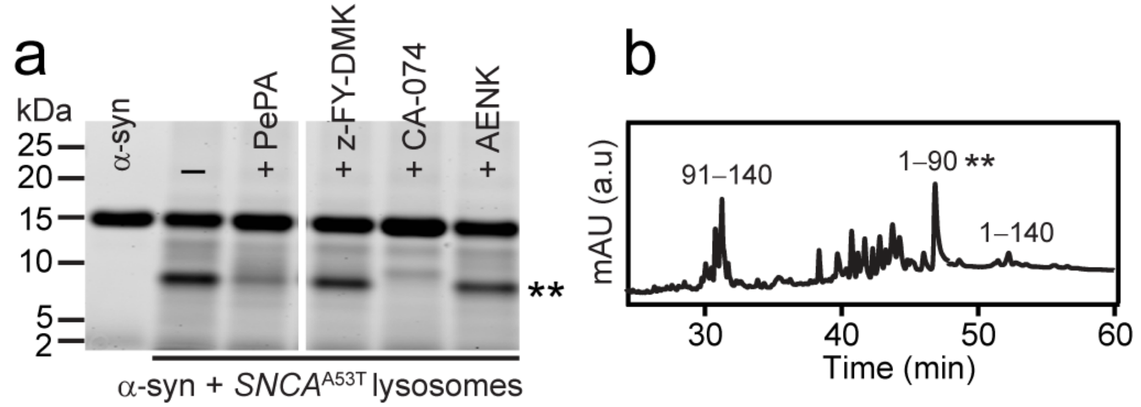


**Figure S4**. Selective inhibitor experiments with lysosomes from *SNCA*$^{A53T}$ mice. (**A**) SDS-PAGE analysis (4–12%) of soluble α-syn (15 μM) in the presence of lysosomal extracts (2.5 μg total protein) from *SNCA*$^{A53T}$ mice for 20 h at pH 5 and 37 °C with the addition of protease inhibitors (1 μM), PePA (CtsD), z-FY-DMK (CtsL), CA-074 (CtsB) and AENK (AEP). Asterisk denotes the 8-kD α-syn truncation. (**B**) LC traces of soluble α-syn (15μM) incubated with lysosomes (5 μg total protein) from *SNCA*$^{A53T}$ mice for 20 h at pH 5 and 37 °C monitored at 210 nm. Peptide fragments found in the corresponding peaks by MS are indicated. All other assigned masses are reported in Table S3.

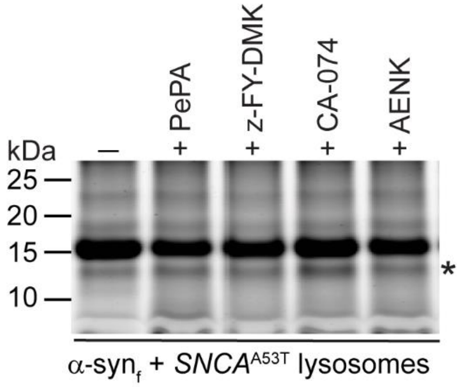


**Figure S5**. SDS-PAGE analysis (4–12%) of preformed α-syn fibrils (15 µM) in the presence of lysosomal extracts from *SNCA*$^{A53T}$ mice (30 µg total protein) for 20 h at pH 5 and 37 °C. The addition of protease inhibitors (1 µM): PePA (CtsD), z-FY-DMK (CtsL), CA-074 (CtsB), and AENK (AEP) are also shown. Asterisk denotes a 12-kD α-syn truncation.

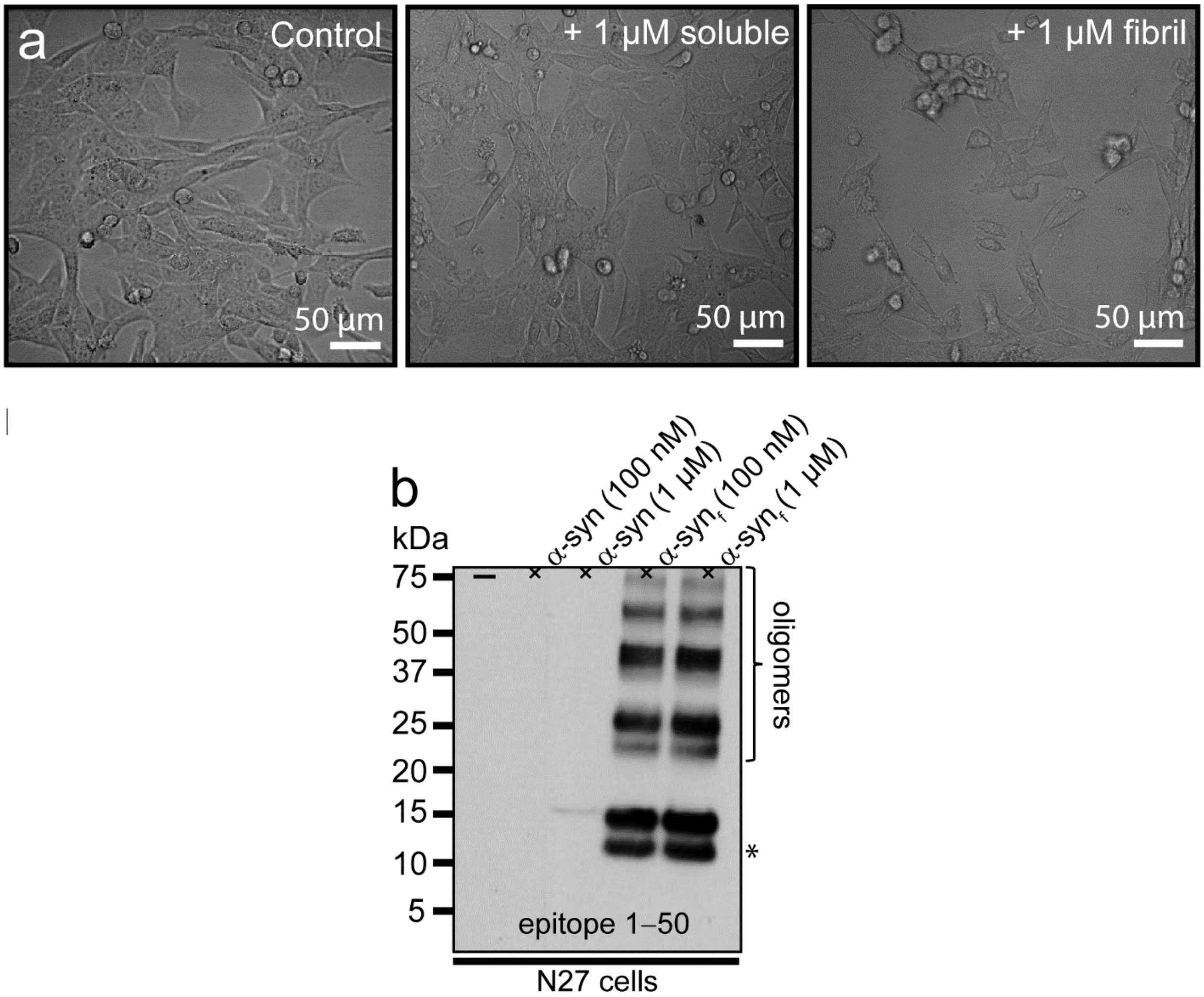


**Figure S6**. Exogenous addition of α-syn to N27 rat dopaminergic neuronal cells. (**A**) Phase contrast images of N27 cells grown in the absence (*left*) and presence of 1 µM soluble α-syn (*middle*) and fibrillar α-syn (*right*) after a 60 h incubation at 37 °C. Soluble and fibrillar α-syn in pH 5 buffer were diluted with media to desired final concentration and added prior to imaging. Images are representative from three independent experiments. (**B**) Western blot analysis probed with N-terminal (epitope 1–50) antibody of N27 cell lysates after 48 h feeding of soluble and fibrillar α-syn (100 nM and 1 µM). Asterisk denotes 12-kDa band.

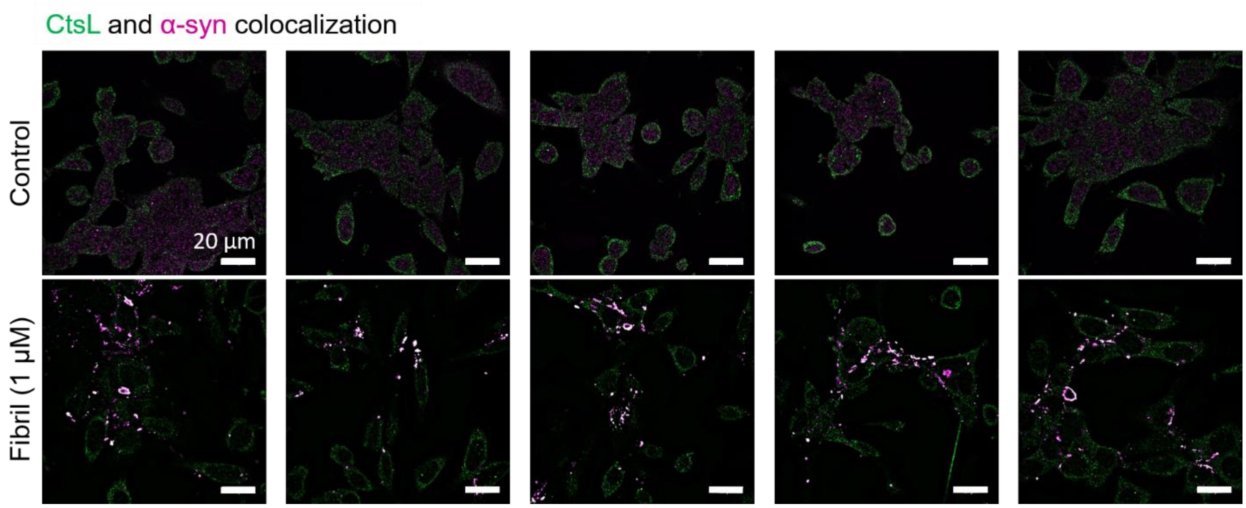


**Figure S7.** Gallery of immunofluorescence images of N27 rat dopaminergic cells co-stained with α-syn and CtsL. Untreated cells (*top*) after 48 h of growth compared to those with exogenously added 1 μM α-syn$_f$ (*bottom*). α-Syn (*magenta*) was stained with an N-terminal rabbit monoclonal antibody, while endogenous CtsL (*green*) was detected using a mouse monoclonal antibody. Scale bar is 20 μm.

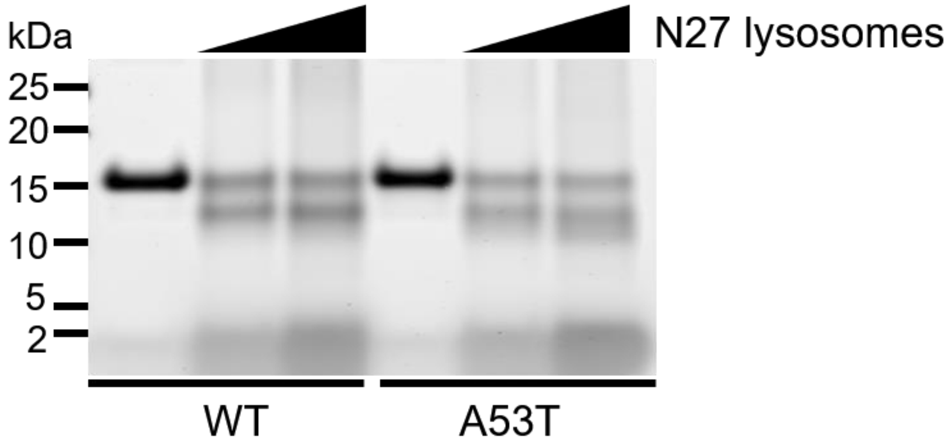


**Figure S8.** SDS-PAGE (4–12%) analysis of N-terminally acetylated wild-type (*left*) and A53T (*right*) α-syn fibrils (15 µM) incubated in the presence of lysosomal extracts (4 and 10 µg) from N27 cells at pH 5 for 20 h and 37 °C. For ease of comparison, the data for WT from Figure 5d are also are shown again.

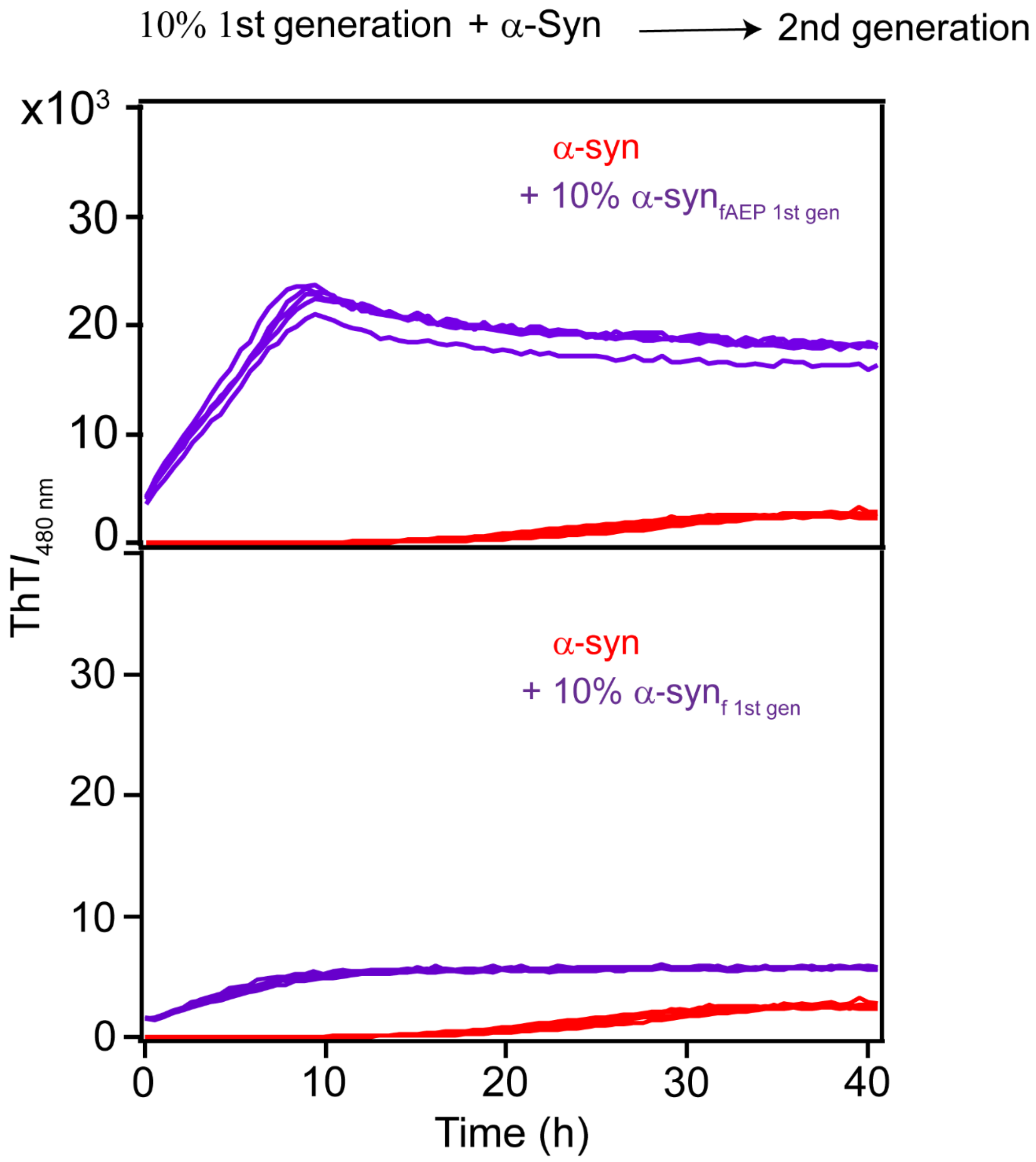


**Figure S9.** Aggregation kinetics monitored by ThT ($\lambda_{obs}$ = 480 nm) of α-syn (50 µM) at pH 7.4 in the absence (red) and presence of 10% (purple) 1st generation α-syn$_{AEP}$ (*top*) and α-syn$_{f}$ (*bottom*) seeds.

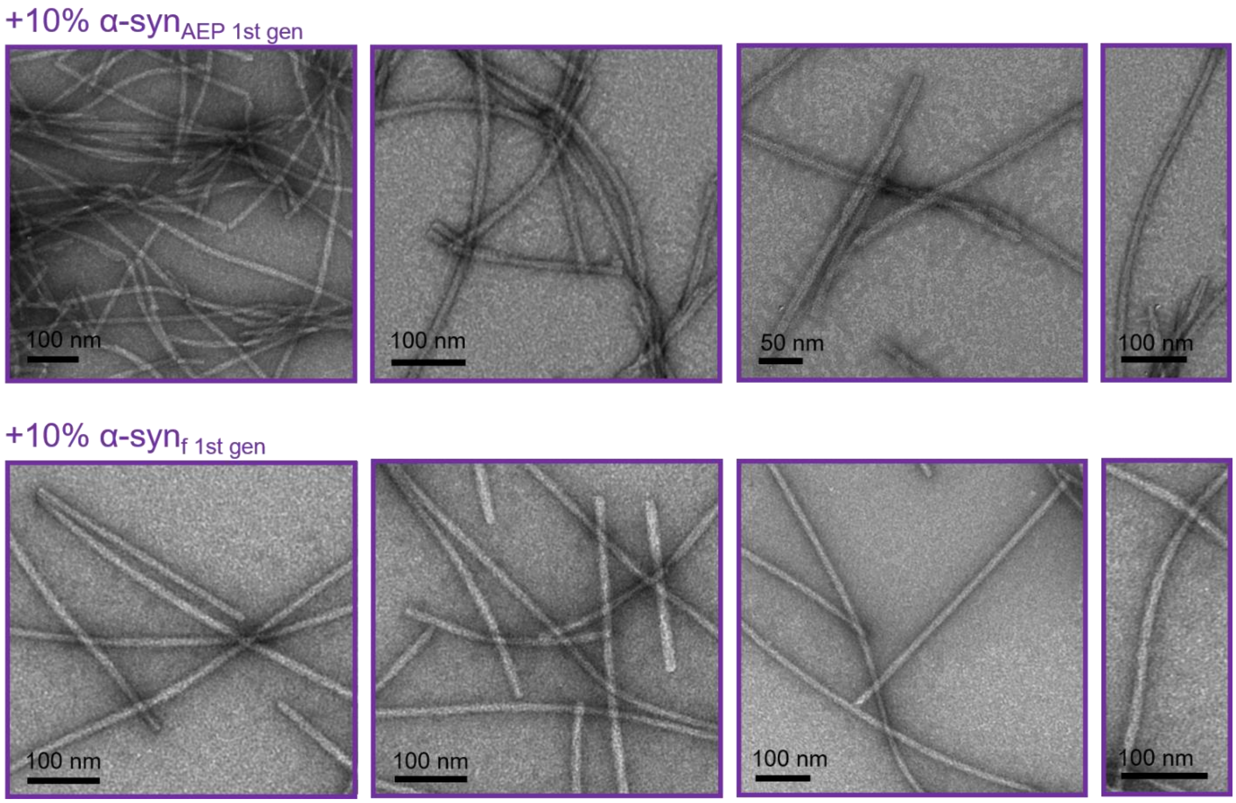


**Figure S10**. Representative TEM images taken of second round seeding experiments of α-syn (50 μM) with 10% (purple) 1st generation of α-syn$_{AEP}$ (*top*) and α-syn$_{f}$ (*bottom*) samples.
